# Prevalence of associated renal risk in type 2 diabetes mellitus in the United Arab Emirates

**DOI:** 10.1111/1753-0407.70038

**Published:** 2024-12-19

**Authors:** Mustafa Jamal Ahmed, Omer Ali, Saf Naqvi, Aisha Ahmed, Waseem Omar

**Affiliations:** ^1^ Kidney Health Services Imperial College London Diabetes Centre Abu Dhabi United Arab Emirates; ^2^ Endocrinology Imperial College London Diabetes Centre (ICLDC) Abu Dhabi United Arab Emirates; ^3^ Faculty of Medicine Pavol Jozef Šafárik University Košice Slovakia

Dear Editor,

Type 2 diabetes mellitus (T2DM) is a chronic metabolic disorder and has become a global health challenge, projected to affect approximately 780 million people by 2045.^1^, ^2^ T2DM is linked to several micro and macrovascular complications.^3^, ^4^ A high prevalence of chronic kidney disease (CKD, 4%–20%) and cardiovascular disease (CVD, 6%–27%) has been observed in patients with T2DM.^5^, ^6^, ^7^, ^8^ The pathophysiological interactions among diabetes, CKD, and CVD are defined as cardiovascular–kidney–metabolic (CKM) syndrome by the American Health Association (AHA).^9^ Managing CKM syndrome involves addressing several risk factors glycated hemoglobin (HbA1c), BP, higher low‐density lipoprotein (LDL) cholesterol, body mass index (BMI), low estimated glomerular filtration rate (eGFR) and high albumin creatinine ratio (ACR).^9^, ^10^, ^11^ Guidelines from Kidney Disease: Improving Global Outcomes (KDIGO, 2024) help in assessing the stage and severity of CKD based on eGFR and ACR.^12^

In recent years, United Arab Emirates (UAE) has experienced an increased risk of metabolic syndromes due to a shift toward less healthy lifestyle habits.^13^, ^14^ The present study aimed to explore the prevalence of T2DM‐associated renal risk in UAE population. The risk factors and T2DM treatment modalities were also evaluated in high and very high‐risk patients. This cross‐sectional study collected data from 33 524 T2DM patients aged ≥18 years registered at the Imperial College Diabetic Center across the seven Emirates. Critical parameters such as age, sex, BMI, systolic and diastolic BP, eGFR, ACR, Hb1Ac, and LDL were collected from the patients. Additionally, data on treatment modalities, SGLT‐2, GLP‐1, RASi, and Finerenone, for treating T2DM were recorded. Statistical analyses were performed to understand the stage of CKD based on KDIGO heat map to find frequency and percentage of patients at high and very high‐risk of CKD.

The study highlighted prevalence of T2DM (53.43%) in the Emirati population and was found to be concurrent with earlier studies.^15^, ^16^, ^17^ Among these patients, the overall prevalence of renal risk was segregated based on eGFR and ACR values (KDIGO heat map). As per eGFR values, 24.25% of population had eGFR values between 60 and 89, while 7.74% T2DM patients exhibited eGFR values less than 60, indicating increased renal risk (Figure 1A). These results are consistent with earlier studies in China, Italy, and United States.^18^, ^19^, ^20^ The Albuminuria (ACR values between 3.5 and 30) was observed in approximately 18% of T2DM patients while severely increased albuminuria (ACR between 30 and 1000) was observed in 5.23% of T2DM patients (Figure 1B). Similar percentage of albuminuria patients has been reported in earlier studies.^21^, ^22^ The patients at high and very highrisk were evaluated for risk factors associated with renal risk. A significant number of patients exhibited suboptimal ranges for risk factors HbA1c, BMI, BP, and LDL, concurrent with earlier study from the Middle East,^23^ which highlights vital role of treatment modalities used in Emirati population. RASi, followed by SGLT2, were the highest administered drugs in high and very high‐risk patients (Figure 1D). The study highlighted that RASi and SGLT2 have significant role in T2DM treatment in UAE. Further studies are warranted to assess the combined effect of all four treatment modalities in T2DM patients.

**FIGURE 1:**
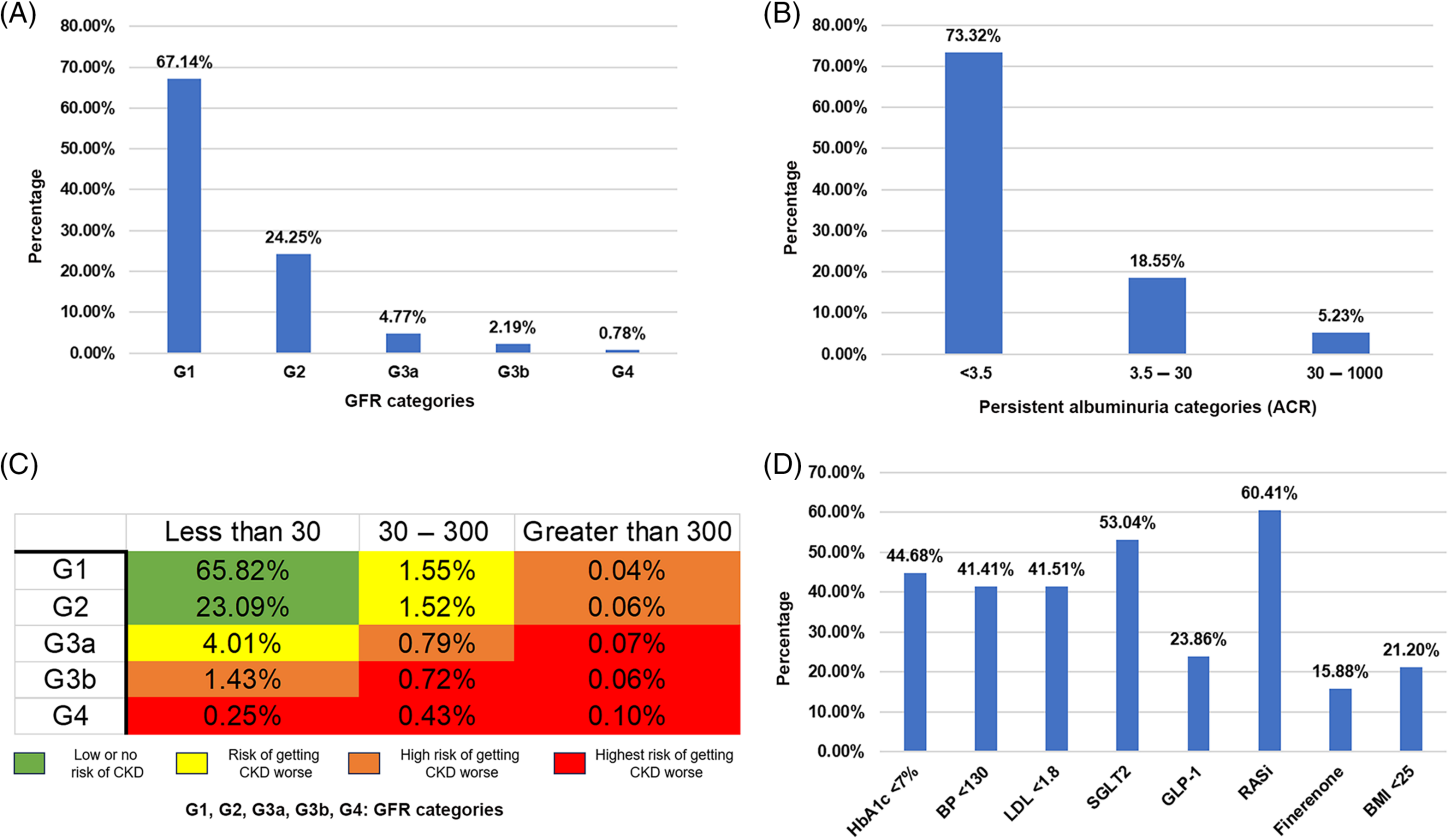
(A) Graph representing the percentage of T2DM patients based on GFR categories. (B) Graph representing the percentage of T2DM patients with ACR values. (C) KDIGO Heat map representing T2DM patients among the Emirati population who are at low, moderate, high, and very high risk of CKD. (D) Graph showing the percentage of T2DM patients with various assessing factors and treatment modalities who are at high and very high‐risk of CKD. ACR, albumin to creatinine ratio; BMI, body mass index; BP, blood pressure; CKD, chronic kidney disease; GFR, glomerular filtration rate; GLP‐1, glucagon‐like peptide; HbA1c, glycated hemoglobin; KDIGO, Kidney Disease: Improving Global Outcomes; LDL, low‐density lipoproteins; RASi, renin angiotensin system inhibitors; SGLT2, sodium‐glucose transport protein 2; T2DM, type 2 diabetes mellitus. GFR categories = G1: Normal or high, G2: Mildly decreased, G3a: Mildly to moderately decreased, G3b: Moderately to severely decreased, G4: Severely decreased; Persistent albuminuria categories (ACR) = <3.5: Normal to mildly increased, 3.5–30: Moderately increased, 30–1000: Severely increased.

## AUTHOR CONTRIBUTIONS

Mustafa Jamal Ahmed was responsible for the study's conceptualization, design, data curation, formal analysis, and drafting of the initial manuscript. Omer Ali, Saf Naqvi, Aisha Ahmed, and Waseem Omar contributed to the critical review, data interpretation, and manuscript editing.

## CONFLICT OF INTEREST STATEMENT

The authors declare no conflicts of interest.
